# Notch signaling pathway is a potential therapeutic target for extracranial vascular malformations

**DOI:** 10.1038/s41598-018-36628-1

**Published:** 2018-12-20

**Authors:** Reema B. Davis, Kristy Pahl, Nicholas C. Datto, Scott V. Smith, Carrie Shawber, Kathleen M. Caron, Julie Blatt

**Affiliations:** 1 Departments of Cell Biology and Physiology, University of North Carolina at Chapel Hill, Chapel Hill, NC USA; 2 Pediatrics (Division of Pediatric Hematology Oncology), University of North Carolina at Chapel Hill, Chapel Hill, NC USA; 3 Surgical Pathology, University of North Carolina at Chapel Hill, Chapel Hill, NC USA; 4 Pathology and Laboratory Medicine (Translational Pathology Laboratory), University of North Carolina at Chapel Hill, Chapel Hill, NC USA; 5 Department of Obstetrics and Gynecology, Columbia University, New York, NY USA

## Abstract

Notch expression has been shown to be aberrant in brain arteriovenous malformations (AVM), and targeting Notch has been suggested as an approach to their treatment. It is unclear whether extracranial vascular malformations follow the same patterning and Notch pathway defects. In this study, we examined human extracranial venous (VM) (n = 3), lymphatic (LM) (n = 10), and AV (n = 6) malformations, as well as sporadic brain AVMs (n = 3). In addition to showing that extracranial AVMs demonstrate interrupted elastin and that AVMs and LMs demonstrate abnormal α-smooth muscle actin just as brain AVMS do, our results demonstrate that NOTCH1, 2, 3 and 4 proteins are overexpressed to varying degrees in both the endothelial and mural lining of the malformed vessels in all types of malformations. We further show that two gamma secretase inhibitors (GSIs), DAPT (GSI-IX) and RO4929097, cause dose-dependent inhibition of Notch target gene expression (*Hey1*) and rate of migration of monolayer cultures of lymphatic endothelial cells (hLECs) and blood endothelial cells (HUVEC). GSIs also inhibit HUVEC network formation. hLECs are more sensitive to GSIs compared to HUVEC. GSIs have been found to be safe in clinical trials in patients with Alzheimer’s disease or cancer. Our results provide further rationale to support testing of Notch inhibitors in patients with extracranial vascular malformations.

## Introduction

Vascular malformations are developmental abnormalities of venous, arterial, capillary or lymphatic vessels which grow with an individual. These can be purely of one type of vessel (e.g., venous [VM] or lymphatic malformations [LM]) or can involve more than one type (e.g., venolymphatic malformations [VLM] or arteriovenous malformations [AVM]^1^. Morbidities are significant such as pain, infection, pulmonary emboli, bleeding, cosmetic concerns, psychosocial issues, and even death. Current treatments include supportive care with compression garments, and interventions such as sclerotherapy, embolization, and surgical debulking or resection. There has been a growing interest in medical management of vascular malformations^2^. The mammalian target of rapamycin (mTOR) inhibitor, rapamycin (Sirolimus), is the most commonly used drug, but does not result in complete resolution of lesions^3^. While generally well-tolerated, its results often are not permanent, and life-long treatment may be needed. Thus, there is a need for other medications.

Notch proteins (Notch 1–4) are a family of receptors that are important in cell differentiation in virtually all tissues. The Notch pathway governs arteriovenous specification leading to distinction of arteries from veins^4^, and paradoxically both loss- or gain-of-function of Notch receptors have been linked to vascular malformations^4^. Ectopic *Notch1*^5^ or *Notch4*^6–9^ activation in endothelial cells results in AVMs. Importantly, activation of *Notch4* in the endothelium of adult mice results in AVMs in organs, including liver, skin, uterus and brain^7^, and AVM formation was reversible upon loss of *Notch4* transgene expression. Likewise, it has been shown that *NOTCH1* signaling is activated in human brain AVMs^9,10^. Knockdown of Notch1 in endothelial cells (ECs) has been found to decrease LEC proliferation and migration^11,12^. Different results may reflect tissue-, species-, and developmental stage-specific differences in the role of Notch signaling, but are all consistent with a role for Notch in aberrant angiogenesis. Several investigators have suggested targeting Notch as an approach to treating AVMs.

In mammals, the Notch signaling pathway is triggered by binding of Notch proteins to any of four activating ligands (Delta-like [Dll1, Dll4], Jagged [Jag1, Jag2]) which ultimately trigger transmembrane cleavage by a gamma-secretase complex, resulting in release of the Notch intracellular domain (NICD), which activates transcription of downstream target genes such as EphrinB2^13^. This is of particular interest because a number of gamma secretase inhibitors (GSI) have undergone phase I and II clinical trials in adults with Alzheimer’s disease^14,15^ and in children and adults with cancer and related disorders^16–18^. That these drugs have known dosing and acceptable safety profiles makes them good candidates for drug repurposing for patients with other disorders such as vascular malformations.

In this study, we show that extracranial vascular malformations, like brain AVMs, are characterized by diminished and incomplete smooth muscle actin (αSMA) in the vascular smooth muscle cells (VSMC) of AVM, and diminished elastin coverage in the internal elastin lamina (IEL) of AVMs. NOTCH1–4 proteins are variably expressed in a range of these extracranial vascular malformations involving lymphatic, venous and arterial vessels. Moreover, two GSIs–DAPT and RO4929097–caused dose-dependent inhibition of Notch target gene expression (*Hey1*) and angiogenesis of both lymphatic and blood endothelial cells *in vitro*. These results provide additional rationale to support clinical trials of GSIs in patients with vascular malformations.

## Results

### Extracranial vascular malformations have discontinuous elastin fibers in their internal elastic lamina

**Figure 1:**
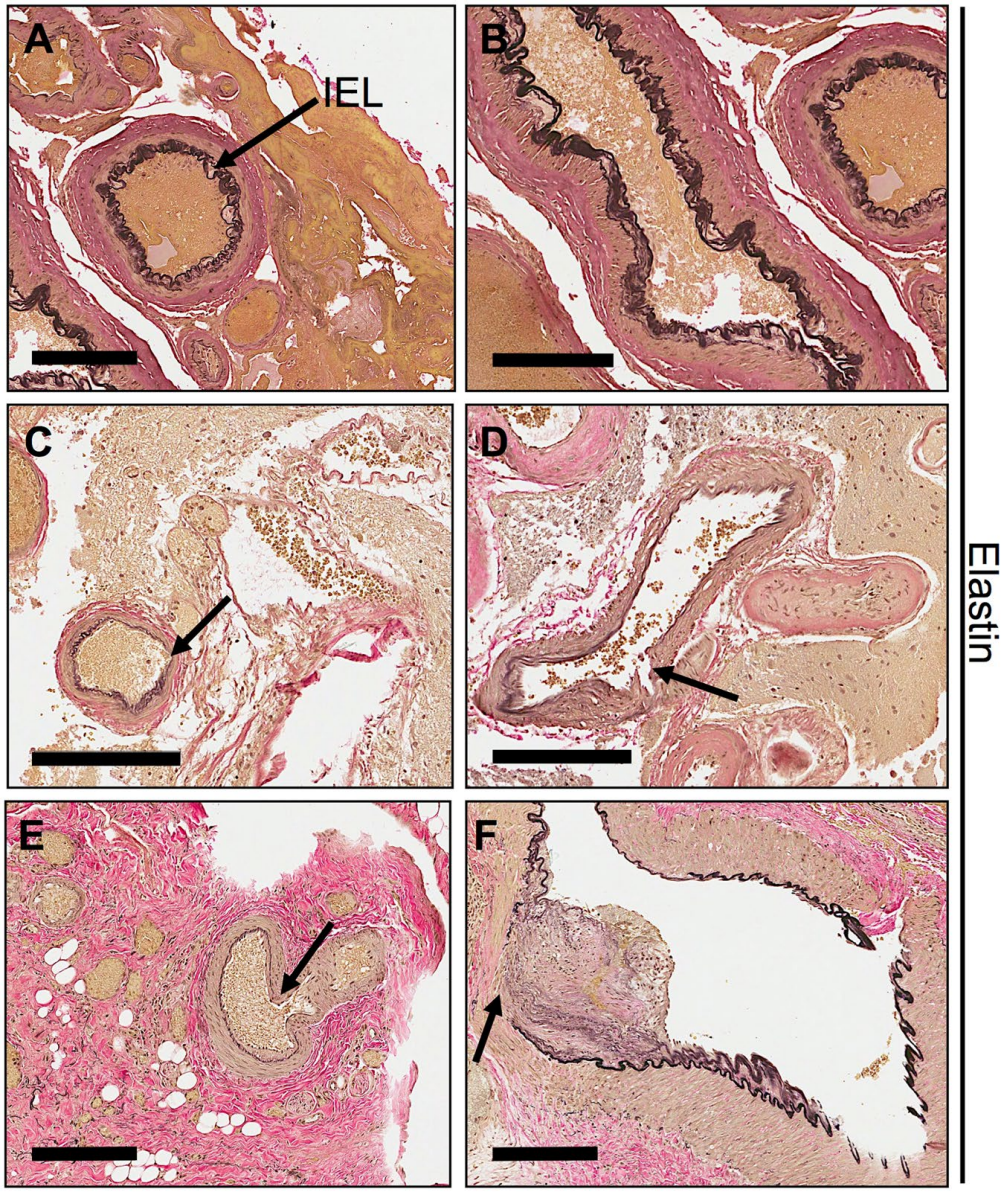
Internal elastic lamina is disorganized in brain and extra-cranial AVMs. (**A**–**F**) Representative images of normal arteries (**A** and **B**) and arteriovenous malformations (**C**–**F**) stained with elastin in tissues from human brain (**C** and **D**), and extra-cranial tissues: thigh (**E**) and nose (**F**). *n* = 2–3 each group. Scale bars, 200 μm. Arrows point to internal elastic lamina (IEL) of major vessels stained with elastin showing either disorganization, diminished, or complete absence of the layer.

Human brain AVMs have disrupted elastin fibers, which compromise the structural integrity of the vessel and mediate arteriovenous malformations^19–21^. While brain elastin content has been well characterized, the elastin in extracranial vascular malformation has not been studied. Therefore, we evaluated tissue samples from human extracranial AVM for elastin fibers in the IEL. Compared to control vessels (Fig. 1A,B), both brain (Fig. 1C,D) and extra-cranial AVMs (Fig. 1E,F) displayed diminished and discontinuous elastin staining in the IEL of major vessels. An additional 3 AVM samples obtained from different regions of the body showed interrupted lamina similar to Fig. 1C–F. Our results indicate that IEL elastin levels are not only abnormal in brain AVMs, but also in extracranial AVMs.

### Active NOTCH1 is expressed in abnormal human extracranial vascular channels

**Figure 2:**
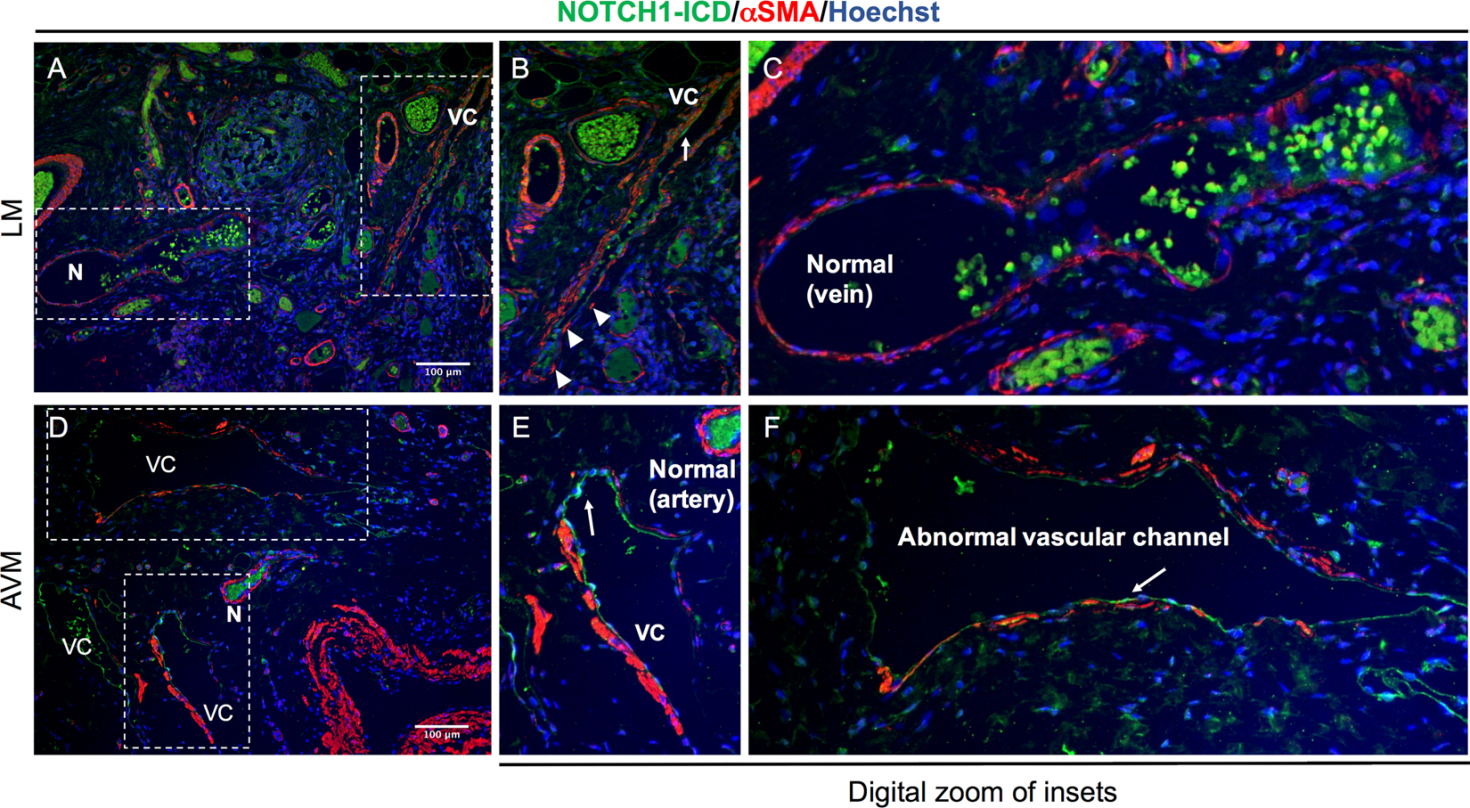
Active NOTCH1 is aberrantly expressed in abnormal vascular channels. (**A**–**F**) Representative images of normal (N) and abnormal vascular channels (VC) stained with smooth muscle marker (αSMA) and Notch1 intracellular domain (NOTCH1-ICD) in tissues from human lymphatic malformation (LM, Fig. **A**–**C**) and arteriovenous malformation (AVM, Fig. **D**–**F**). *n* = 2 each group. Scale bar, 100 μm. Arrows point to endothelial lining of vessels prominently expressing NOTCH1-ICD. Boxed regions are shown as digitally zoomed insets to the right.

Normal arteries have a uniform layer of αSMA-positive mural cells around them, and in contrast, normal lymphatic capillaries have a single endothelial layer with no mural cell coverage. However, arteries involved in AVMs are characterized by incomplete αSMA coverage and malformed lymphatic vessels gain mural cell coverage around them^22^ (Fig. 2). Due to the role of Notch signaling in normal and pathological vascular development and arterial venous specification, we determined the expression of active NOTCH1 intracellular domain (NOTCH1-ICD) in abnormal vascular channels (VC). Compared to minimal expression in normal (N) vessels with complete αSMA (Fig. 2C), NOTCH1-ICD was clearly seen in all the abnormal VC (Fig. 2A,B,D–F) within the same sections. There was no NOTCH1-ICD expression overlapping with αSMA staining indicating that NOTCH1 is primarily activated in the endothelium of malformed vessels. An additional 8 tissues from a broad spectrum of LM subtypes were stained with an antibody against the extracellular domain of NOTCH1, and the lymphatic endothelial protein, PODOPLANIN (Supplemental Table 1). NOTCH1 expression was observed in the endothelium of 6 of these LM samples, but not in normal lymphatic vessels.

### NOTCH2 and 3 are expressed in endothelial and mural cells surrounding vascular malformations

**Figure 3:**
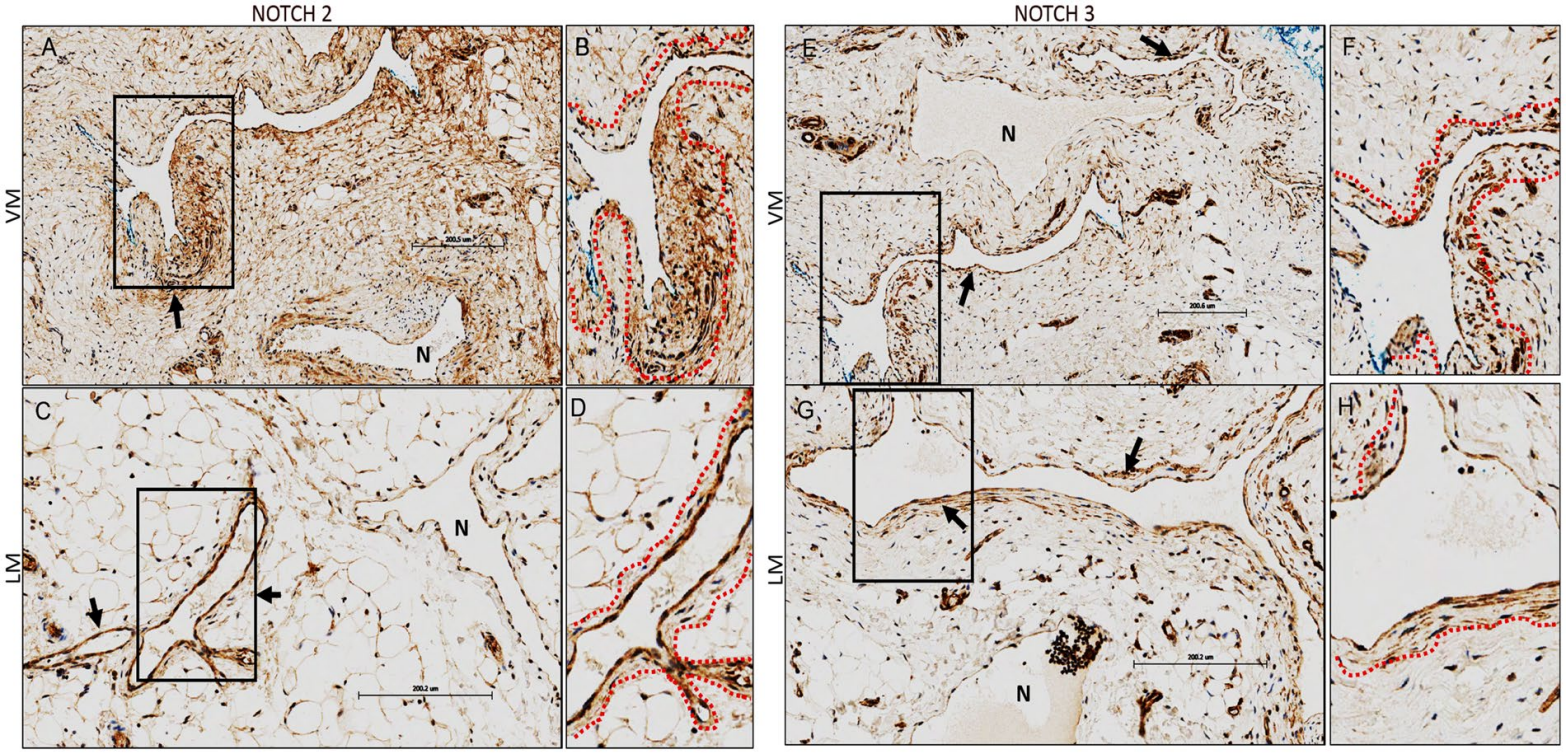
NOTCH2 and 3 are prominently expressed in the endothelial and some mural cells lining venous and lymphatic malformations. (**A**–**H**) Representative images of NOTCH2 (**A**–**D**) and NOTCH 3 (**E**–**H**) in human extra-cranial venous (VM) and lymphatic malformations (LM). *n* = 2–4 each group. B, D, and F,H represent digitally zoomed image of boxed insets in A,C and E,G respectively. Dotted red lines highlight regions of NOTCH2 and 3 expression in mural cells surrounding malformations. Scale bar, ~200 μm. Arrows point to the endothelial and mural cells lining malformed vessels prominently expressing NOTCH2 and 3. “N” represents a normal vessel within the same section.

Studies have shown that NOTCH2 is minimally expressed, while NOTCH3 is overexpressed in human brain vascular malformations and a combined loss of one allele of *Notch1* with *Notch3* deletion results in CADASIL (a genetically mediated arteriopathy primarily of cerebral arteries and arterioles) and AVMs in mice^21,23^. Therefore, we examined NOTCH2 and NOTCH3 expression in extracranial LM and VM. Our results show NOTCH2 and NOTCH3 clearly are expressed in endothelial and mural cells lining both VMs and LMs, and appear to be increased compared to normal vessels (N) within the same field (Fig. 3A–D). NOTCH2 was expressed in cells outside of the vasculature in venous malformations and it was distinctly expressed mainly in vascular channels of LMs (Fig. 3A,C). NOTCH3 staining was obvious in both LM and VM (Fig. 3B,D). Our results suggest that unlike in brain AVMs, NOTCH2 and NOTCH3 are expressed in extracranial lymphatic and venous malformations, and in the endothelial and to some extent even mural cell layer surrounding the malformations.

### NOTCH4 shows variable expression in malformed vessels of venous, lymphatic and arteriovenous origin

**Figure 4:**
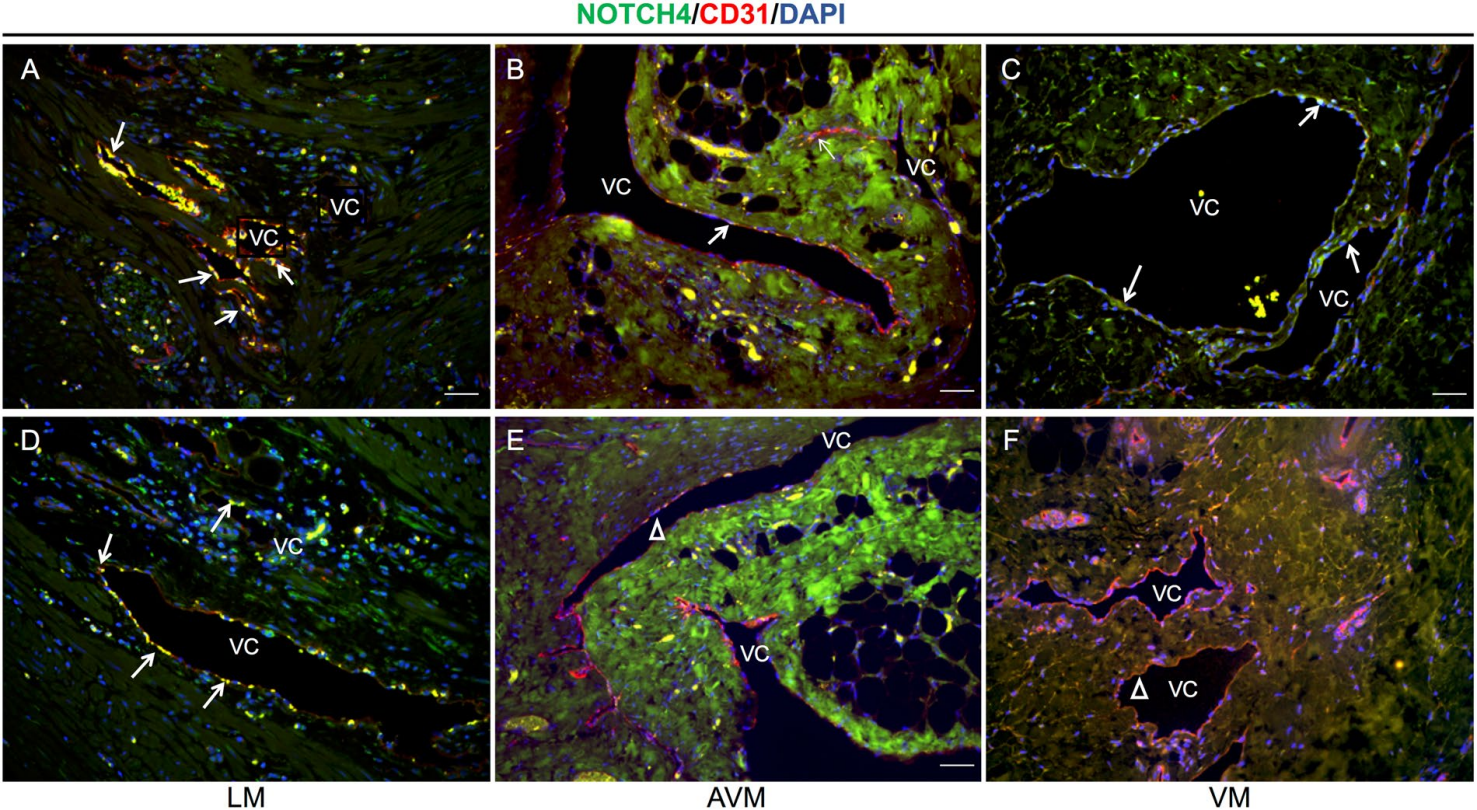
NOTCH4-ICD shows irregular expression in abnormal vascular channels. (**A**–**F**) Representative images of abnormal vascular channels (VC) stained with endothelial marker (CD31) and NOTCH4-ICD in tissues from human lymphatic malformation (LM, Fig. **A** and **D**), arteriovenous malformation (AVM, Fig. **B** and **E**), and venous malformation (VM, Fig. **C** and **F**). *n* = 2 each group. Scale bar, 100 μm. Arrows point to endothelial lining of vessels showing the presence or absence of NOTCH4-ICD.

Staining with an antibody against NOTCH4-intracellular domain (NOTCH4-ICD) demonstrated varied expression across different types of malformations. Lymphatic malformation sections showed strong NOTCH4-ICD expression in CCD31+ endothelial cells lining the abnormal vascular channels consistent with active NOTCH4-ICD signaling (Fig. 4A,D). NOTCH4-ICD expression in AVM sections was minimal with occasional high expression in a few endothelial cells (Fig. 4B,E). Tissue from VM showed weak and interrupted NOTCH4-ICD expression in CD31 weak vessels and was absent from CD31+ abnormal vascular channels (Fig. 4C,F). Collectively, these data suggest a link between the misexpression of Notch family members and NOTCH1 and NOTCH4 activation in human extracranial vascular malformations of all types.

### DAPT and RO4929097downregulate *Hey1* expression in blood and lymphatic endothelial cells without altering human cell viability

**Figure 5:**
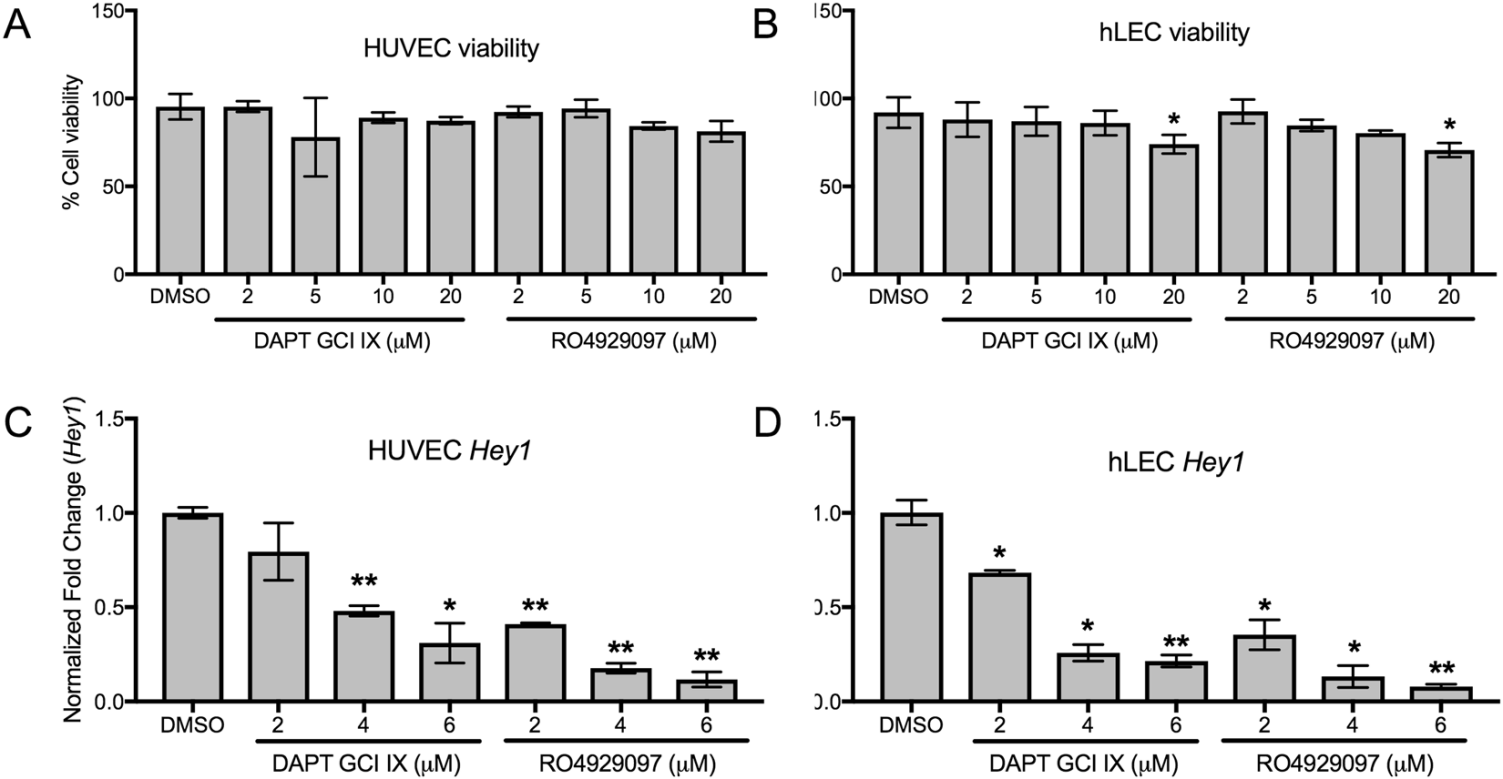
DAPT and RO4929097 downregulate downstream Notch target *Hey1* without altering cell viability. (**A**,**B**) Measurement of HUVEC (**A**) and hLEC (**B**) cell viability after GSI treatments. Quantitative data are represented as mean ± SEM. *n* = 3 for HUVEC and hLEC. Significance was determined by 2-tailed, type 2 Student’s t test, *P < 0.05. (**C**,**D**) Relative expression of downstream Notch target gene *Hey1* in GSI-treated HUVEC (**C**) and hLEC (**D**). Quantitative data are represented as mean values of fold change over DMSO control ± SEM. *n* = 4 for each cell line. *Gapdh* and *β-actin* were used as housekeeping control. Significance was determined by 2-tailed, type 2 Student’s *t* test, *P < 0.05, **P < 0.01.

First, we confirmed that the GSIs in concentrations used in this study are successful in inhibiting the Notch signaling pathway in human blood (HUVECs) and lymphatic endothelial cells (hLECs) without altering their viability. Cell viability was not significantly different compared to untreated controls at concentrations less than 20 μM on either cell type. 20 μM of DAPT and RO4929097 reduced hLEC viability by almost 30% without significant changes observed at lower concentrations (Fig. 5A). Neither drug altered HUVEC cell viability at any of the concentrations tested (Fig. 5B). The expression of the NOTCH target gene *Hey1* was significantly downregulated in both cell lines. DAPT downregulated *Hey1* expression in HUVECs beginning at 4 μM, while 2 μM of RO4929097 was sufficient to downregulate *Hey1* (Fig. 5C). hLECs appeared more sensitive to GSIs, with both drugs causing inhibition at concentrations as low as 2 μM (Fig. 5D).

### γ-secretase inhibitors can effectively inhibit angiogenesis of both human blood and lymphatic cultured endothelial cells

**Figure 6:**
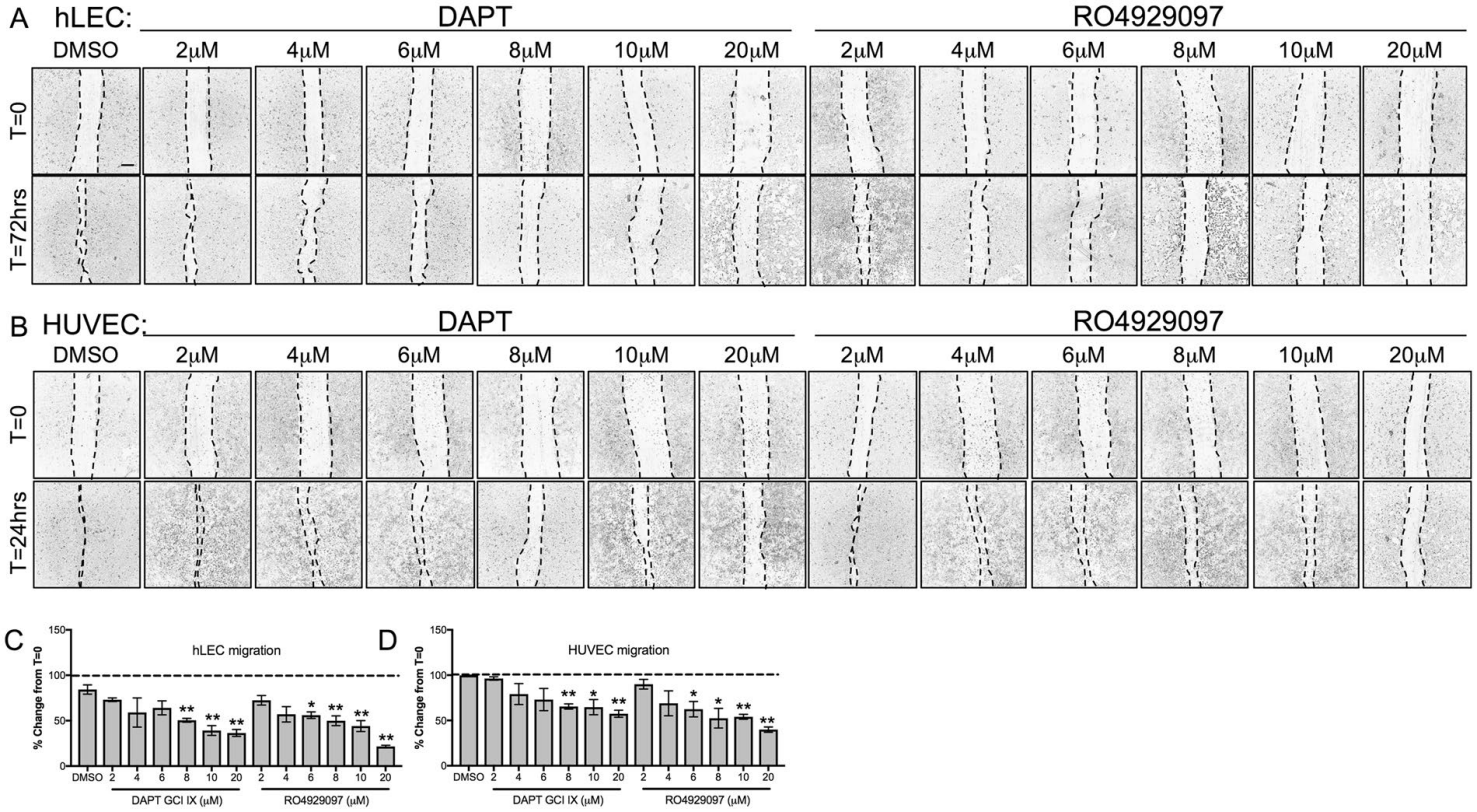
GSIs block cellular migration (**A**,**B**) Control DMSO or GSI treated hLEC (**A**) at 72 hrs and HUVEC (**B**) at 24 hrs post-scratch. (**C**,**D**) Migration from the time of scratch (T = 0) for hLEC (**C**) and HUVEC (**D**) was measured. Quantitative data are represented as mean ± SEM. *n* = 4 for HUVEC and *n* = 3 for hLEC. Significance was determined by 2-tailed, type 2 Student’s t test, *P < 0.05, **P < 0.01. Scale bar, 200 μM.

While the use of DAPT in the inhibition of migration and proliferation of blood endothelial cells has been well characterized^16–19^, to our knowledge there are no studies for the use of DAPT or RO4929097 on lymphatic endothelial cells. Therefore, we investigated the effectiveness of both these GSIs on the rate of migration of monolayer cultures of hLEC and HUVEC cells (Fig. 6A,B). 8 μM of DAPT reduced hLEC migration to about 50% and HUVEC migration to about 35% while RO4929097 was more effective at 6 μM in both cell types (Fig. 6C,D). RO4929097 reduced migration in hLECs by greater than 50% at lower concentrations compared to HUVECs.

**Figure 7:**
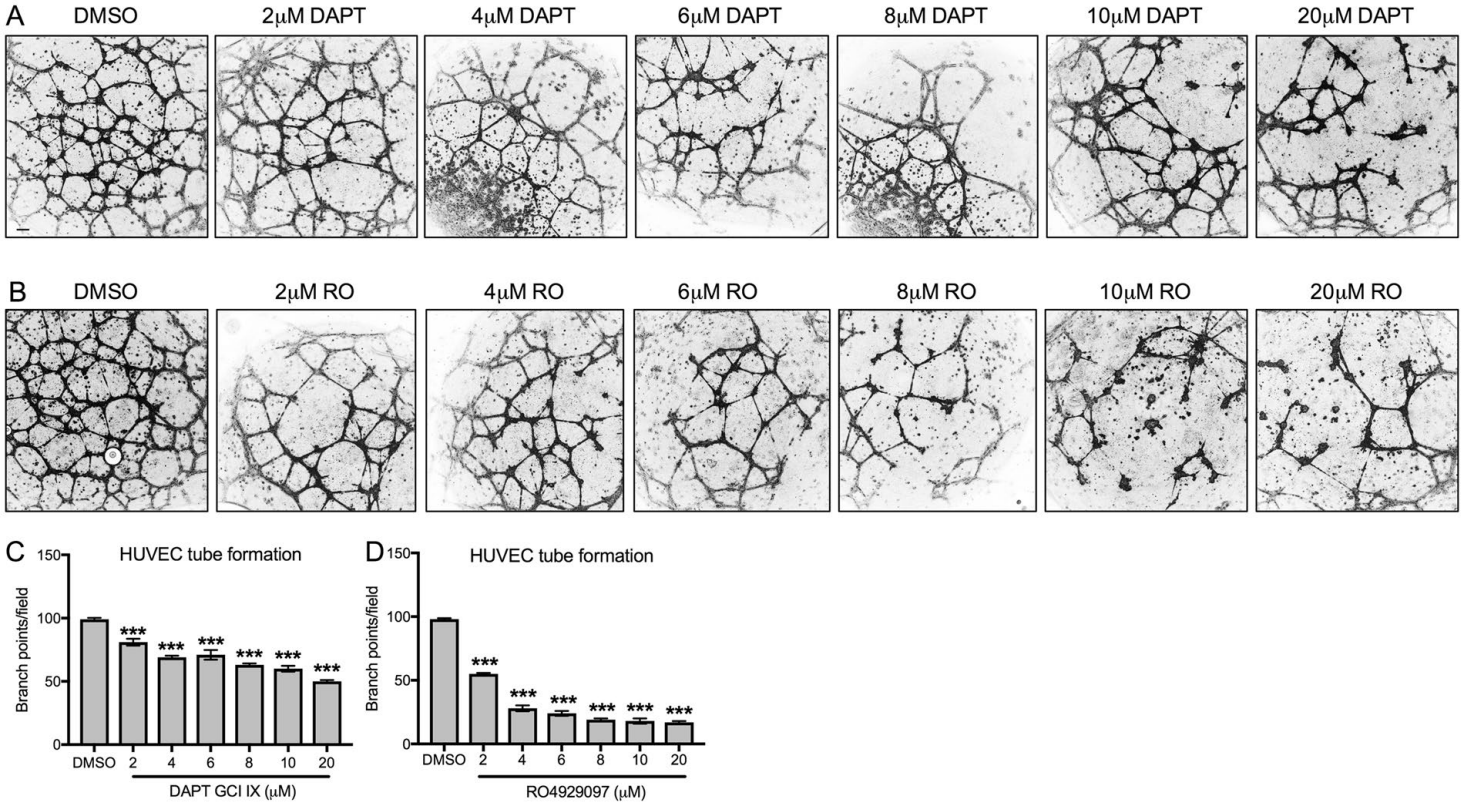
GSIs block tube formation (**A**,**B**) Representative images of tubes formed by control DMSO treated or GSI DAPT (**A**) or RO4929097 (**B**) treated HUVEC at 24 hrs. (**C**,**D**) Branch points per field were quantified. Quantitative data are represented as mean ± SEM. *n* = 4 for DAPT treated (**C**) or RO treated (**D**) HUVEC. Significance was determined by 2-tailed, type 2 Student’s t test, ***P < 0.001. Scale bar, 100 μM.

We determined the effect of GSIs on network formation of cultured human endothelial cells. Evaluation of tube formation by HUVEC in a matrigel matrix after treatment with increasing doses of DAPT and RO4929097showed that network formation was dramatically reduced even with the lowest concentration (2 μM) of the GSIs (Fig. 7A,B). The number of branch points seen per field, an operational read-out of network formation assays, was significantly reduced with all concentrations of DAPT and RO4929097, with the latter being more potent in inhibiting network formation (Fig. 7C,D). Taken together, our results demonstrate that, HUVECs and hLECs are susceptible to both GSIs, and that lymphatic endothelial cells are more susceptible to GSIs compared to HUVECs.

## Discussion

In this study, we examined a wide range of human extracranial vascular malformations involving lymphatic, venous and arterial vessels and demonstrate that Notch proteins are expressed in each type. Although our results are qualitative, activated NOTCH1-ICD is more apparent in endothelial cells lining extracranial LM, VM and AVM compared to normal vessels within the same tissue. Full length NOTCH1 expression is observed in the endothelium of a majority of the LMs independent of whether the lesion was a focal (cystic LM) or multi-focal (generalized lymphatic anomaly/Gorham Stout). The broader expression of full length NOTCH1 than NOTCH1-ICD in the LM endothelium is consistent with the mechanism of NOTCH signaling, where Notch active cells are surrounded by ligand-expressing Notch inactive cells. NOTCH2 and NOTCH3 were expressed in endothelial and mural cells lining both VMs and LMs, and appeared to be increased compared to what we observed in normal vessels. NOTCH4-ICD was present in LM, but was sparsely expressed in both brain and extracranial AVM and VM. Our results suggest that Notch signaling plays a key role in human extracranial lymphatic, venous, and arteriovenous malformations and adds to the previously focused information on NOTCH overexpression in brain AVMs. We also found that, similar to those in the brain, extracranial AVMs showed interrupted αSMA and elastin layer surrounding malformed vessels.

The GSIs, DAPT and RO4929097, were effective in inhibiting angiogenesis in Notch-expressing HUVECs. RO4929097, which has been used in human clinical trials^17^, was more potent on a molar basis than DAPT, commonly used *in vitro*. Furthermore, hLECs were more susceptible to Notch inhibition than HUVECs. Our results are consistent with previous findings of GSI inhibition of HUVEC migration and tube formation^24–26^ and add to the information with comparative analysis between two GSIs across two different endothelial cells lines. Although GSIs are known to be direct inhibitors of Notch, it also is possible that these drugs have effects downstream of Notch, e.g., on EphrinB2, which also is gamma secretase-processed when activated. Nonetheless, taken together our results present a strong impetus for targeting the Notch signaling pathway in the management and treatment of extracranial lymphatic, venous and arteriovenous malformations with GSIs. Combined loss of one allele of *Notch1* and global deletion of *Notch3* has been linked with retinal AVMs in mice^23^. Notch2, while not described to be expressed in normal peripheral vascular cells, has a role in renal and eye capillary development^27^. Notch-mediated communication between vascular endothelial cells and smooth muscle cells is critical during normal vascular development. Notch signaling is also important to the pathogenesis of vascular disease, and lies both upstream and downstream of critical vascular and lymphatic growth signals such as VEGF, EphB4/ephrinB2 and adrenomedullin/CLR signaling^28–30^. Data on the role of Notch in human vasculature are limited, but patients with CADASIL are known to have missense mutations in *Notch3* or *Jag1*^31^. Patients with Alagille syndrome, a pleiotropic developmental disorder involving multiple organs, have demonstrated mutations in the Notch ligand *Jag1*^32^. Our results support and extend several studies which have identified NOTCH1, 3 and 4 overexpression in AVMs of the central nervous system (CNS) in humans^10,21,33^. Possible tissue-, species-, and developmental stage-specific differences in the role of Notch signaling underline the need to study both inhibition and up-regulation in human vascular malformations.

Our observations are of particular interest because several GSIs already have been through clinical trials, both in children and adults. Tolerated doses and schedules for oral administration have been well defined, particularly in older patients. Although lack of efficacy in Alzheimer’s disease, the initial target population, led to shut-down of development of this class of drugs by the pharmaceutical industry, some efficacy was suggested in leukemias, solid tumors and in benign but locally aggressive desmoid tumors^14–18^. The maximum concentrations observed in pharmacokinetic studies of RO4929097 in patients with cancer were in the range of concentrations which we studied^34^. Renewed interest in making drug available for one or more of these indications or for other rare disorders seems likely in the near future. We studied only one of 5 GSIs which have been used in humans. Preclinical testing of the other four drugs of this class will be of interest. Additional preclinical studies will be needed to detail the signaling pathways involved in development and progression of vascular malformations. However, we suggest that these can proceed concurrent with pilot phase II clinical trials of Notch inhibitors in patients with these disorders.

## Materials and Methods

### Patient samples and IRB approval

We confirm that all experimental protocols were carried out in accordance with relevant guidelines and regulations. All experimental protocols were approved by the following IRB committee: A database (approved by the University of North Carolina [UNC] School of Medicine’s Institutional Review Board [IRB]) of all patients with vascular anomalies (ICD9 747.64, 74; 7.60, 759.9, 228) seen at UNC January 2000-January 2016 by members of our multi-disciplinary Vascular Anomalies Clinic was compiled (JB, KP). Entries were reviewed for diagnostic accuracy, patient demographics, the availability of relevant archived biopsies, location of the anomaly, and treatment prior to biopsy. For human studies at UNC, LM, VM, and AVM tissue samples were collected from archived surgical specimens and samples were de-identified. A waiver of consent and waiver of HIPPA authorization were approved by the **UNC IRB (IRB 15-1423)**. For human studies at Columbia, de-identified LM tissues were collected from discarded surgical specimens. As protected health information (PHI) was neither stored nor disclosed to the researchers, tissues were collected under exemption by **Columbia University IRB (AAAA7338)** and thus, there was no verbal or written consent required.

After obtaining an IRB waiver of approval, de-identified samples based on the database (VM [n = 3], LM [n = 2], extracranial AVM [n = 6], brain AVM [n = 3]) were obtained in paraffin blocks from UNC’s Department of Surgical Pathology. The extra-CNS biopsies came from variable locations including head and neck, chest, abdomen and extremities. An attempt was made to select the most recent specimens from patients who had not had prior sclerotherapy or malformation-directed medications. No patient had received GSIs for any disease. Because of the heterogeneity typical of histologic samples of vascular malformations, archival blocks were reviewed (SVS) to optimize sectioning of the part of the block most involved by the vascular malformation. An additional 8 LM samples were available from Columbia P&S.

### Cell Culture

Dermal human lymphatic endothelial cells (hLECs) from human neonates (HMVEC-dLyNeo-Der, Lonza CC-2812) and human umbilical venous endothelial cells (HUVEC, Lonza CC-2517) were used for *in vitro* assays (below) within 8 passages and maintained in EGM-2MV bullet kit (Lonza CC3202) and EGM bullet kit media respectively.

### *In vitro* Assays

#### Cell viability

To examine the effect of GSI on cell viability, hLEC and HUVEC cultures were incubated with effects of graded concentrations [2, 4, 6, 8, 10, 20 μM] of DAPT (Selleckchem, S2215) or RO4929097 (Selleckchem, S1575) [2–20 uM] for 24–72 hours. The percentage of viable cells was determined using the Countess automated cell counter (Thermofisher, C10227). Briefly, 80–90% confluent HUVEC and hLEC cultures were trypsinized, washed, and 10 μL of cell sample was mixed with an equal amount of 0.4% trypan blue stain and loaded onto the counting chamber.

#### Scratch migration assay

Migration assay was performed as per Liang *et al*.^35^. HUVECs and hLECs were grown to confluence in 24 well dishes, and then scratched with a 200 ul pipette tip. After scratching, the wells were rinsed with 1XPBS to remove non-adherent cells and then treated with control DMSO or increasing concentrations [2, 4, 6, 8, 10, 20 μM] of DAPT or RO4929097. Four fields per well were imaged at T = 0 hrs and at T = 24 hrs post-scratch or T = 72 hrs for HUVECs and hLECs respectively using an Olympus IX-81 inverted microscope equipped with a QImaging Retiga 4000 R camera at 4X magnification. The percent change in migration was calculated by measuring the open area of the scratch at the above mentioned time-points (ImageJ). Results shown are representative of four independent experiments with HUVECs and three for hLECs.

#### Tube formation assay

This assay was performed based on the protocol by Arnaoutova and Kleinman^36^. Briefly, HUVEC cells were serum-starved after reaching 70–80% confluence, trypsinized, washed and plated at similar concentrations in a Growth Factor Reduced Matrigel Matrix (BD Biosciences 356230) coated 96-well plate and submerged in growth medium containing either control DMSO or increasing concentrations (2, 4, 6, 8, 10, 20 uM) of DAPT or RO4929097.

### RNA and quantitative RT-PCR

RNA was extracted from cultured HUVEC or hLEC cells either 24 hrs or 72 hrs post treatment respectively using TRIzol reagent (Ambion 15596026) followed by DNase (Promega M6101) treatment and cDNA prepared using iScript (BioRad 170-8890). Quantitative RT-PCR was done on StepOnePlus (ABI) using TaqMan Gene Expression Master Mix (TheroFisher Scientific 4369016). Gene expression was assessed using human Single-tube assays (Thermo Fisher Scientific/Applied Biosystems): *GAPDH* (4310884E), *ACTB* (Hs99999903_m1) and *HEY1* (Hs01114113_m1). Comparative ΔΔC_T_ method was used to analyze relative gene expression with ExpressionSuite Software (Thermo Fisher Scientific). Expression was normalized to housekeeping controls *GAPDH* and *ACTB*.

### Immunofluorescence

Paraffin sections were stained as per Davis *et al*.^28^. Briefly, they were rehydrated first followed by 20 minutes boiling for antigen retrieval in 10 mM Sodium citrate, 0.05% Tween 20, pH 6.0. They were then permeabilized in 1% triton X-100/PBS for 20 minutes, and blocked in 5% NDS. Sections were incubated with primary antibodies, including mouse anti-smooth muscle alpha actin (1:200, Sigma-Aldrich A4700) and rabbit anti-activated NOTCH1 (1:100, Abcam ab8925) and NOTCH4 (1:100, Abcam ab33163) primary antibodies for 2 hours at room temperature. Sections were washed and incubated in secondary antibodies including donkey anti-rabbit Cy3 (Jackson ImmunoResearch 711-225-152), donkey anti-mouse Cy2 (Jackson ImmunoResearch 715-545-151), and Bisbenzimide H 33258 Hoechst (Sigma-Aldrich B1155) at 1:250 at room temperature for 1 hour. The tissue sections were mounted in Prolong gold (Life technologies P36934). Rabbit polyclonal antibodies against NOTCH2 (ab8926) and NOTCH3 (ab60087) were from Abcam (Cambridge, MA). Immunohistochemistry (IHC) carried in the Bond fully-automated slide staining system (Leica Bicrosystems Inc. Vist, CA). Slides were deparaffinized in Bond dewax solution (AR9222) and hydrated in Bond wash solution (AR9590). Antigen retrieval for all targets was performed at 1000 C in Bond-epitope retrieval solution 1 pH6.0 (AR9961) for 20 min. After pretreatment NOTCH2 (1:500) and NOTCH3 (1:200) were applied for 30 min. Detection was performed using Bond polymer refine detection system (DS9800). Stained slides were dehydrated and coverslipped. Positive and negative controls (no primary antibody) were included for each antibody. Elastin special stain was done at the UNC Animal Histopathology Core (AHC). Photomicrographs were reviewed (JB, KP, SVS) to confirm the types of cells (endothelium, muscle, pericyte) which were stained by each antibody. NOTCH staining was evaluated semi-quantitatively by comparing that of abnormal vessels within the malformation with positive and negative control tissues and with normal intra-lesional vessels. Abnormal vessels were categorized as showing more, less, or the same amount and intensity of staining than the controls. A rabbit polyclonal NOTCH4-ICD generated at Columbia P&S and goat polyclonal full-length NOTCH1 (1:100 R&D Systems AF1057) staining were previously described^37^.

### Image Acquisition

Images for the assays were taken on an Olympus microscope with cellSens software. Immunofluorescence images were acquired on a Nikon E800 fluorescence microscope with a Hammamatsu Orca CCD camera with Metamorph software (Molecular Devices Corp.). NOTCH2, 3, and elastin stained sections were digitally imaged (20x objective) in the Aperio ScanScope XT using line-scan camera technology (Leica Biosystems). Digital images were stored in the Aperio eSlide Manager software.

### Statistical analysis

All experiments were performed 3 or more times with 3 technical triplicates per assay and data are represented as a mean with SD or SEM. Significance was determined by Student *t* test (tail = 2, type = 2) with *P < 0.05, **P < 0.01 and ***P < 0.001 considered significant.

## Electronic supplementary material

Notch signaling pathway is a potential therapeutic target for extracranial vascular malformations

## Data Availability

Data will be made available upon acceptance.
