# Author Correction: Notch signaling pathway is a potential therapeutic target for extracranial vascular malformations

**DOI:** 10.1038/s41598-020-58751-8

**Published:** 2020-01-30

**Authors:** Reema B. Davis, Kristy Pahl, Nicholas C. Datto, Scott V. Smith, Carrie Shawber, Kathleen M. Caron, Julie Blatt

**Affiliations:** 1 Departments of Cell Biology and Physiology, University of North Carolina at Chapel Hill, Chapel Hill, NC USA; 2 Pediatrics (Division of Pediatric Hematology Oncology), University of North Carolina at Chapel Hill, Chapel Hill, NC USA; 3 Surgical Pathology, University of North Carolina at Chapel Hill, Chapel Hill, NC USA; 4 Pathology and Laboratory Medicine (Translational Pathology Laboratory), University of North Carolina at Chapel Hill, Chapel Hill, NC USA; 5 Department of Obstetrics and Gynecology, Columbia University, New York, NY USA

Correction to: *Scientific Reports* 10.1038/s41598-018-36628-1, published online 20 December 2018

This Article contains errors. In Figure 5c and 5d, the concentrations provided are incorrect; the correct Figure 5 appears below as Figure 1.

**Figure 1:**
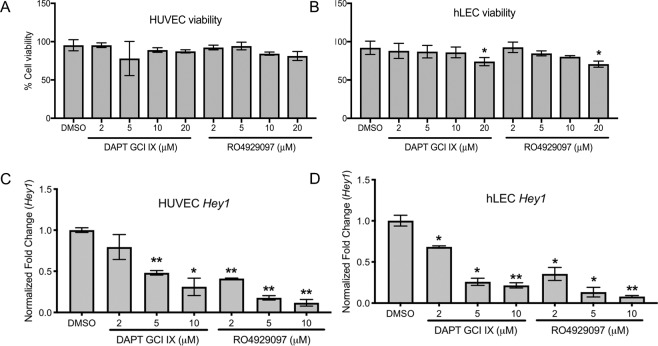
DAPT and RO4929097 effectively inhibit Notch signaling without altering cell viability. (**A,B**) Measurement of HUVEC (**A**) and hLEC (**B**) cell viability after GSI treatments. Quantitative data are represented as mean ± SEM. *n* = 3 for HUVEC and hLEC. Significance was determined by 2-tailed, type 2 Student’s t test, *P < 0.05. (**C,D**) Relative expression of downstream Notch target gene *Hey1* in GSI-treated HUVEC (**C**) and hLEC (**D**). Quantitative data are represented as mean values of fold change over DMSO control ± SEM. *n* = 4 for each cell line. *Gapdh* and *β-actin* were used as housekeeping control. Significance was determined by 2-tailed, type 2 Student’s *t* test, *P < 0.05, **P < 0.01.

In Figure 6, several duplicate images were inadvertently introduced, and the concentrations listed are incorrect. A re-analyzed and re-assembled Figure 6, with the correct information, is provided below as Figure 2.

**Figure 2:**
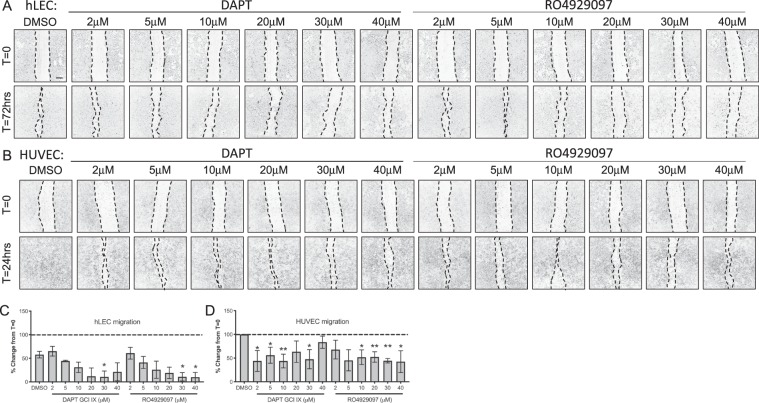
GSIs block cellular migration (**A**,**B**) Control DMSO or GSI treated hLEC (**A**) at 72 hrs and HUVEC (**B**) at 24 hrs post-scratch. (**C**,**D**) Migration from the time of scratch (T = 0) for hLEC (**C**) and HUVEC (**D**) was measured. Quantitative data are represented as mean ± SEM. *n* = 4 for HUVEC and *n* = 3 for hLEC. Significance was determined by 2-tailed, type 2 Student’s t test, *P < 0.05, **P < 0.01. Scale bar, 200 μM.

As a result, in the Results section under subheading “γ-secretase inhibitors can effectively inhibit angiogenesis of both human blood and lymphatic cultured endothelial cells”,

“8 μM of DAPT reduced hLEC migration to about 50% and HUVEC migration to about 35% while RO4929097 was more effective at 6 μM in both cell types (Fig. 6C,D)”.

should read:

“30 μM of DAPT reduced hLEC migration by 90% and HUVEC migration by about 50%; HUVEC migration was significantly reduced at 2 μM. RO4929097 had significant effect on hLEC starting 30 μM and 10 μM on HUVECs (Fig. 6C,D)”.

And, under subheading “DAPT and RO4929097downregulate Hey1 expression in blood and lymphatic endothelial cells without altering human cell viability”,

“DAPT downregulated Hey1 expression in HUVECs beginning at 4 μM, while 2 μM of RO4929097 was sufficient to downregulate Hey1 (Fig. 5C)”.

should read:

“DAPT downregulated Hey1 expression in HUVECs beginning at 5 μM, while 2 μM of RO4929097 was sufficient to downregulate Hey1 (Fig. 5C)”.

In addition, there are the following typographical errors in the Results section under subheading “DAPT and RO4929097downregulate Hey1 expression in blood and lymphatic endothelial cells without altering human cell viability”,

“Neither drug altered HUVEC cell viability at any of the concentrations tested (Fig. 5B)”

should read:

“Neither drug altered HUVEC cell viability at any of the concentrations tested (Fig. 5A)”

And, in the same section:

“20 μM of DAPT and RO4929097 reduced hLEC viability by almost 30% without significant changes observed at lower concentrations (Fig. 5A)”

should read:

“20 μM of DAPT and RO4929097 reduced hLEC viability by almost 30% without significant changes observed at lower concentrations (Fig. 5B)”

Finally, in the Methods section, under subheading “Cell viability”,

“Graded concentrations [2, 4, 6, 8, 10, 20 μM] of DAPT (Selleckchem, S2215) or RO4929097 (Selleckchem, S1575) [2–20 uM] for 24–72 hours.”

should read:

“Graded concentrations [2–20 μM] of DAPT (Selleckchem, S2215) or RO4929097 (Selleckchem, S1575) [2–20 μM] for 24–72 hours”.

And under the subheading “Scratch migration assay”,

“After scratching, the wells were rinsed with 1XPBS to remove non-adherent cells and then treated with control DMSO or increasing concentrations [2, 4, 6, 8, 10, 20 μM] of DAPT or RO4929097”

should read:

“After scratching, the wells were rinsed with 1XPBS to remove non-adherent cells and then treated with control DMSO or increasing concentrations [2, 5, 10, 20, 30, 40 μM] of DAPT or RO4929097”

The main conclusions of the Article are unaffected by these changes.

