# Supplementary material for: Notch signaling pathway is a potential therapeutic target for extracranial vascular malformations
Source: Sci Rep. 2018 Dec 20;8:17987. doi: 10.1038/s41598-018-36628-1 (PMC6302123; doi:10.1038/s41598-018-36628-1)
Supplement: Supplementary file 1 — Notch signaling pathway is a potential therapeutic target for extracranial vascular malformations [file 41598_2018_36628_MOESM1_ESM.pdf]

Notch signaling pathway is a potential therapeutic target for extracranial vascular malformations

Reema B. Davis, Ph.D.[1], Kristy Pahl, M.D.[2], Nicholas C. Datto[1], Scott V. Smith, M.D.[3,4], Carrie Shawber, Ph.D.[5], Kathleen M. Caron, Ph.D.[1], Julie Blatt, M.D.[2*]

Supplementary information

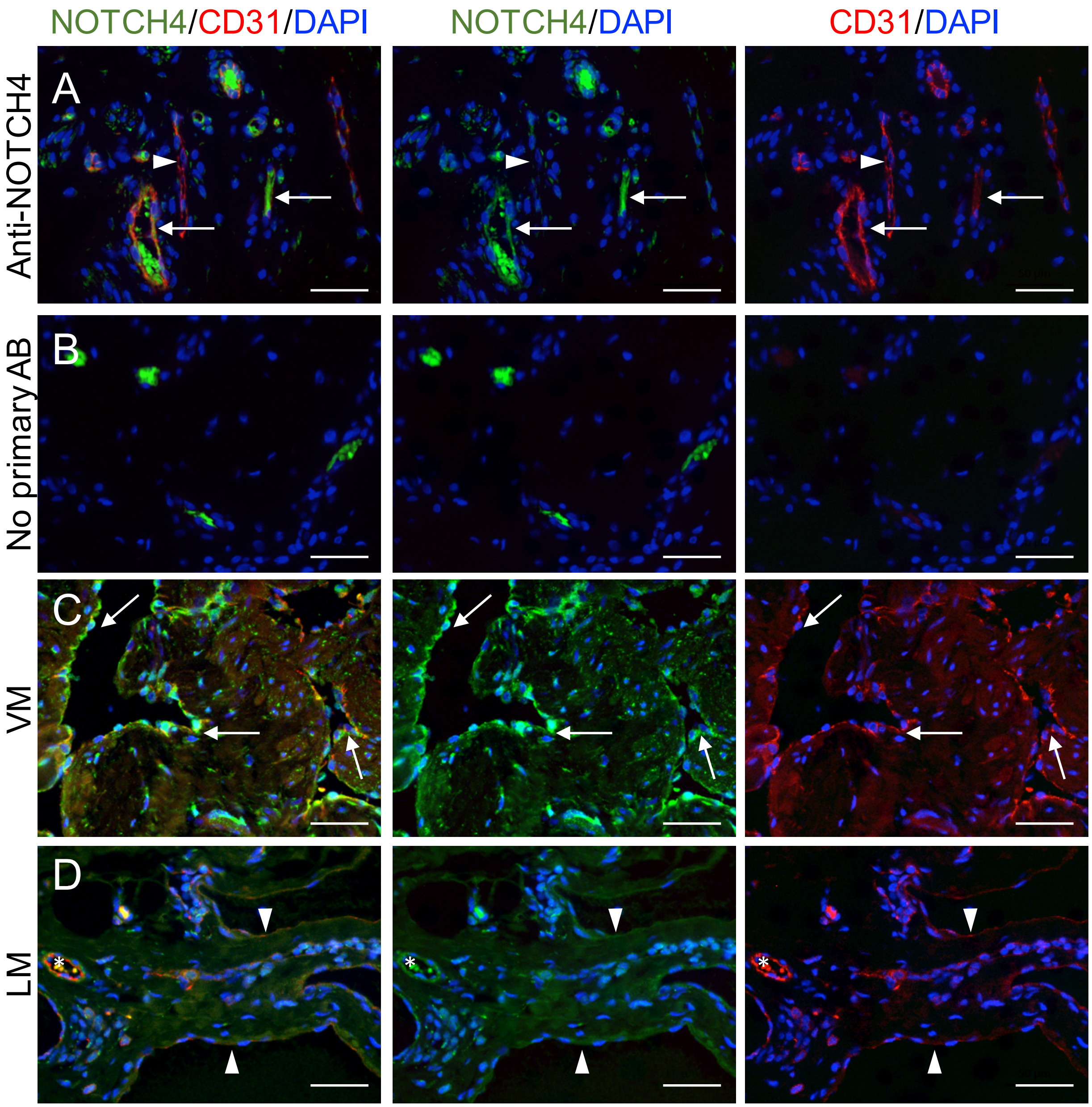

Supplemental Figure 1. Vascular NOTCH4 expression in control and vascular malformation tissues. A,B) NOTCH4 is expressed in CD31+ endothelial cells in control neonatal skin. A) Notch4 antibody, B) no primary antibody. White arrows marker the NOTCH4 expressing vessels. White arrowheads mark absence of NOTCH4 expression. C,D) NOTCH4 expression is variable in the CD31+ endothelial of vascular malformations. C) VM, D) LM. White arrows marker the NOTCH4 expressing vessels. White arrowheads mark absence of NOTCH4 expression. Asterisk marks normal artery in LM section. Scale bar 50 um.

Supplementary table 1: Organ/tissues of samples used in this study

LM	VM	AVM
neck	thigh mass	posterior thigh
left axilla	left leg	nose
right axilla	finger	shoulder
right arm	finger	brain
pancreatic	thigh	brain
left posterior scalp	upper lip	right forearm/hand
intra-abdominal	right cheek	brain
left foot		face
mediastinal/thymus		finger
right axilla		
right neck		

Supplementary table 2: NOTCH1 expression in the subtypes of malformations

LM subtype	NOTCH1
Cervicofacial macro/microcystic LM	Positive
Lung Generalized Lymphatic Anomaly	Positive
Lower limb dermis Gorham'Stout	Positive
Buttock dermis Generalized Lymphatic Anomaly	Negative
Perinium dermis Generalized Lymphatic Anomaly	Positive
Mesenteric macrocystic LM	Positive
Upper limb dermis macro/microcystic LM	Negative
**VM subtype**	**NOTCH1**
Thigh mass	Positive
Left leg	Negative
Finger	Positive
**AVM subtype**	**NOTCH1**
Posterior thigh	Positive
Nose	NA
Shoulder	NA
Face	Positive
Finger	Positive
Right forearm/hand	Negative
